# What makes an intervention dyadic? Introducing the DYADIC meta-framework to Describe Your focAl Dyadic Intervention Components

**DOI:** 10.1093/abm/kaaf102

**Published:** 2026-02-03

**Authors:** Corina Berli, Urte Scholz, James M Allen, Sally Di Maio, Patrick S Höhener, Nina Knoll, Aleksandra Luszczynska, Monique S Nakamura, Jeffry A Simpson, Gertraud Stadler, Karoline Villinger, Lea O Wilhelm, Alexander J Rothman

**Affiliations:** Department of Health Psychology and Behavioral Medicine, Institute of Psychology, University of Bern, CH-3012 Bern, Switzerland; Department of Psychology, University of Zurich, 8050 Zurich, Switzerland; Department of Psychology, University of Zurich, 8050 Zurich, Switzerland; School of Health and Wellbeing, University of Glasgow, Glasgow, G12 8TB, United Kingdom; University Outpatient Clinic, Medical School Berlin, 13359 Berlin, Germany; Department of Psychology, University of Zurich, 8050 Zurich, Switzerland; Department of Education and Psychology, Freie Universität Berlin, 14195 Berlin, Germany; Center for Applied Research on Health Behavior and Health (CARE-BEH), SWPS University, Wroclaw, 53238 Wroclaw, Poland; Lyda Hill Institute for Human Resilience, University of Colorado at Colorado Springs, Colorado Springs, CO 80918, United States; Department of Community Health and Health Behavior, University at Buffalo, Buffalo, NY 14214, United States; Department of Psychology, University of Minnesota, Minneapolis, MN 55455, United States; Center for Prevention, Health and Human Sciences, Gender in Medicine Research Unit, Charité Universitätsmedizin Berlin, 13353 Berlin, Germany; Department of Psychology, University of Zurich, 8050 Zurich, Switzerland; Department of Psychology, Columbia University, New York, NY 10027, United States; Department of Psychology, Medical School Berlin, 14197 Berlin, Germany; Department of Education and Psychology, Freie Universität Berlin, 14195 Berlin, Germany; Department of Psychology, University of Minnesota, Minneapolis, MN 55455, United States

**Keywords:** dyadic interventions, behavior change, meta-framework, close relationships, dyad, behavior change techniques, mechanisms of action

## Abstract

**Background:**

Engaging in health behaviors often occurs within a social context. This recognition has led to a notable growth in intervention approaches designed explicitly to involve a “close other,” often referred to as dyadic interventions. Yet, there has been surprisingly little discussion of what makes an intervention dyadic.

**Methods:**

To address this gap, we developed the DYADIC meta-framework (Describe Your focAl Dyadic Intervention Components) based on iterative discussions.

**Results:**

The DYADIC meta-framework delineates 4 dimensions that capture distinct ways an intervention can be dyadic: Who is there? What is done? How does it work? What is the outcome? These features can combine in distinct configurations, such that an intervention may be dyadic in only 1 dimension or across all 4. For each dimension, we propose criteria to distinguish between individual and dyadic operationalizations. The DYADIC meta-framework for dyadic interventions broadens how researchers conceptualize an intervention as dyadic, identifies meaningful ways in which dyadic interventions can differ, and facilitates testing whether different dyadic features uniquely promote behavior change.

**Conclusions:**

Together, these contributions lay the foundation for generating the evidence-based guidance to optimize dyadic intervention design. The framework is designed to be applicable across diverse dyad types (eg, romantic partners, family members, adolescent friends).

## Introduction

There is growing consensus among scholars that health behaviors routinely unfold within social contexts. We eat lunch with coworkers, watch a movie with friends, and manage illness through routines shared with a spouse. Social relationships clearly matter for health,^1^^,^^2^ and this recognition has spurred the development of interventions that actively involve a “close other.”^3^^,^^4^ Interventions engaging 2 people in a close relationship (ie, a dyad) are commonly referred to as *dyadic interventions*, and they come in a variety of forms. For instance, a spouse may be invited to join a partner in a maternal health counseling session,^5^ participate in cardiac rehabilitation,^6^ or send reminders to encourage physical activity.^7^ Or an individual may be asked to collaboratively plan a joint physical activity routine with a family member or friend.^8^

Despite the growing interest in dyadic approaches, there has been surprisingly little discussion about what actually makes an intervention *dyadic.* For example, is an intervention dyadic if 1 person simply sits in the waiting room while their partner receives treatment? Or does it require that both individuals actively work together such as with collaborative planning? Or does it depend on whether the intervention targets dyadic mechanisms of action (MoAs) such as social support or reciprocity, or on whether the intended outcome involves joint behavior change, such as quitting smoking together? These questions point to 4 features that may characterize an intervention as dyadic: (1) the presence of one or both dyad members, (2) the type of intervention technique used, (3) the MoA targeted, and (4) the primary intervention outcome.

While dyadic interventions are comprised of one or more dyadic operationalizations of these features, the rationale for doing so is rarely articulated. Are certain dyadic features more effective than others in stimulating behavior change? Are some combinations of features more effective? Researchers have increasingly turned to dyadic approaches to promote behavior change,^3^^,^^4^ but there has been limited progress in articulating how dyadic interventions differ from one another and from individual or group-based interventions. To advance our understanding of what makes a dyadic intervention effective, a conceptual framework is needed to describe the defining features of dyadic interventions.

### What is a dyadic behavior change intervention?

Since the early 2000s, behavior change interventions have increasingly leveraged the dyad, with systematic reviews and meta-analyses examining their effectiveness across diverse health domains.^3^^,^^4^^,^^9–12^ While findings are somewhat encouraging, meta-analyses have revealed substantial heterogeneity in intervention effects.^4^ One likely contributor to this variability is the absence of a consistent, standardized definition of a dyadic behavior change intervention.^13^ This diversity in conceptualization is reflected in the inclusion criteria across reviews. Some define dyadic interventions as those involving both partners directly in the treatment groups,^11^ whereas others require involvement of self-identified couples, even if treatment is not administered to both partners concurrently.^12^ Still other reviews use criteria such as “couple-based” or “dyadic”^4^^,^^9^ without further specification.

To bring greater clarity, Di Maio et al.^14^ (p. 6) propose using “dyadic interventions” as an umbrella term, defined as interventions that “explicitly address both members of a dyad as part of the intervention, using a range of techniques to target either one or both dyad partners to change at least 1 dyad partner’s health behavior.” This broad definition allows for a continuum, for dyad partners being simply present to actively collaborating, but it does not specify which features make an intervention distinctively dyadic. For example, does it matter if a dyad partner is passively present (eg, waiting outside a session) or actively engaged through techniques, or if the intervention is designed to elicit dyadic MoAs and/or promote a dyadic behavioral outcome?

### The need for a meta-framework

Despite growing interest in characterizing aspects of dyadic interventions,^3^^,^^4^ no systematic framework has been proposed to specify their core features. This limits our understanding of potential sources of variability across these interventions. In a first attempt to do so, DiMaio et al.^14^ and Scholz et al.^13^ identified 4 prototypes that capture different conceptualizations of dyads in interventions: (1) passive presence of a dyad partner (“mere presence”), (2) both dyad partners receiving an individual intervention in the same intervention setting (“parallel”), (3) one dyad partner being instructed to interact with the other (“cross-over”), and (4) both dyad partners working collaboratively (“joint”). While useful as a starting point, these prototypes focused mainly on describing differences in intervention techniques (ie, what is done). However, they fail to capture the myriad of other ways interventions may be dyadic, for instance, what types of mechanisms or outcomes are targeted.

To address this gap, we introduce the Describe Your focAl Dyadic Intervention Components (DYADIC) meta-framework. The DYADIC meta-framework is comprised of 4 key dimensions along which a behavior change intervention can be dyadic. Each dimension is paired with definitions that distinguish between individual and dyadic operationalizations.

The DYADIC framework offers several key contributions: (1) It helps identify and distinguish the meaningful ways in which dyadic behavior change interventions can vary; (2) it broadens conceptualizations of what makes an intervention dyadic and encourages researchers to consider assessing dyadic mechanisms and outcomes; and (3) it enables systematic testing of which dyadic features contribute uniquely to behavior change, which, in turn, should lead to evidence-based guidance for optimizing dyadic intervention design.

In the following, we first provide an overview of the proposed DYADIC meta-framework, then describe each of its dimensions in detail, and finally its implications and future directions.

## The DYADIC meta-framework of dyadic interventions: an overview

**Figure 1:**
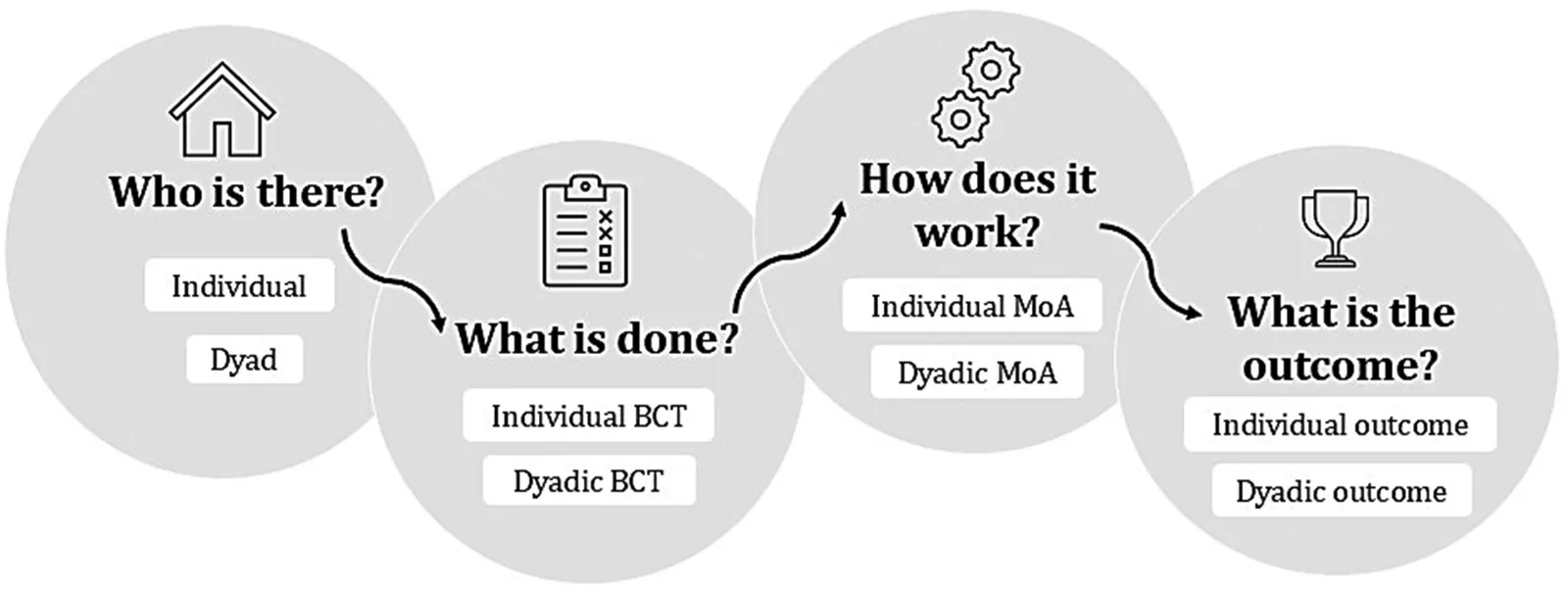
Overview of the DYADIC meta-framework. BCT, behavior change technique; MoA, mechanism of action.

The DYADIC meta-framework identifies 4 core dimensions that distinguish how an intervention engages either individuals or a dyad (Figure 1).

1. Who is there?—Captures who is physically or virtually present during the intervention.
2. What is done?—Specifies the intervention content, specifically the behavior change techniques (BCTs) applied.
3. How does it work?—Describes the underlying MoAs that the intervention targets.
4. What are the outcomes?—Specifies the intended behavioral outcomes of the intervention.

An intervention can be considered dyadic if any one of the 4 dimensions is operationalized at the dyadic level. To illustrate, consider patient A who is recovering from a myocardial infarction and is invited to participate in a rehabilitation program focused on improving diet and physical activity. In a traditional individual-level intervention, only patient A is present during the intervention, which involves the delivery of individual BCTs (eg, self-monitoring) designed to prompt individual MoAs (eg, self-efficacy) and, in turn, improve patient A’s outcomes (eg, diet and exercise routine). The DYADIC meta-framework identifies 4 ways in which this intervention could be modified to become dyadic: (1) A close family member is invited to participate in a session with patient A; (2) the intervention incorporates collaboration between patient A and the family member; (3) the intervention targets dyadic MoAs such as seeking support from a family member; and (4) the intervention promotes improvements in both patient A’s and the family member’s health behaviors. These features can combine in distinct configurations. For example, an intervention might involve the family member’s presence but focus solely on patient A’s behavior, or it might integrate both collaboration and dyadic mechanisms, or even aim for changes in both partners’ behaviors. This example illustrates how varied combinations across the 4 dimensions give rise to a multitude of potential dyadic interventions, each engaging the dyad in different ways.

In the following sections, we elaborate on each dimension, offering definitions to distinguish between individual and dyadic configurations. For an overview of all dimensions and operationalizations, see Table S1.

### Dimension 1: Who is there

Consider a different behavior change intervention in which a pregnant woman receives counseling on maternal health behaviors. As the person whose behavior is the target of the intervention, the pregnant woman can be referred to as the “target person.” Now imagine her partner is invited to accompany her to the session.^5^ Does the mere presence of the partner make this a dyadic intervention? According to prior categorizations,^13^^,^^14^ interventions where a dyad partner is simply present while a target person receives an intervention (“mere presence”) or where both dyad partners receive an individual intervention (“parallel intervention”) qualify as dyadic. These forms are common, comprising roughly one-fourth of dyadic behavior change interventions among couples.^14^ But what exactly does “presence” entail?

The first dimension of the DYADIC meta-framework, *Who is there?,* aims to capture the distinct ways in which a dyad partner may be present and the implications of that presence for classifying an intervention as dyadic. Let us expand the initial example across 3 scenarios: (1) Only the pregnant woman is invited and attends the session alone; (2) the pregnant woman and her partner are both invited to the session, but when the intervention is delivered (eg, ultrasound screening, counselling advice, health check-up), the partner either remains in the waiting room or receives different counselling content in a separate session; and (3) both the pregnant woman and her partner are invited and attend the same session together, sharing exposure to the same content. In both scenarios 2 and 3, the pregnant woman’s partner is present at the clinic and aware that she is receiving counseling, but the degree to which the partner is aware of or engaged with the counseling session differs. To capture this, the framework distinguishes 2 guiding questions for this first dimension:

1. *Who is invited to the intervention?* Is the invitation extended to only one member of the dyad (a target person or their dyad partner) or both? Dyads may be composed of a designated target person who is supposed to change their behavior and their dyad partner, or of 2 dyad partners that are both supposed to change their behavior.
2. *Who is present when the intervention content is delivered?* Is one or both dyad partners present when the intervention content (eg, information, instructions, training or procedure) is delivered?

The 2 guiding questions allow us to systematically differentiate between the 3 intervention scenarios described above (Table 1). If both partners are invited to the intervention, but only one of the partners (either the target person or the partner) is present when the intervention content is delivered, we define this as *dyadic partial presence*. If both dyad partners are invited to the intervention and are in the same space (physically or virtually) so that they hear or see each other and hear or see the same information during intervention delivery, we define this as *shared presence*. Importantly, what the intervention content includes and what tasks have to be completed are not specified here, but is captured by the next dimension (what is done?).

**Table 1:** Individual and dyadic operationalizations of dimension 1, “Who is there?.”

	Labels	Scenario	Example
**Individual**	Solo presence	(1) One member of the dyad (*Target Person* or *Dyad Partner*) is invited to the intervention alone and is the only person present when intervention is delivered.	A pregnant woman is invited to a maternal counselling appointment. She receives counselling regarding maternal health behaviors.
**Dyadic**	Dyadic partial-presence	(2) *Both dyad partners* are invited to the intervention, but either the *Target Person or Dyad Partner* is the only person present when intervention is delivered.	A pregnant woman is invited to a maternal counselling appointment. Her partner is invited to accompany her to the counselling appointment, but… (a) when the intervention is delivered (eg, counselling advice, ultrasound), the partner is not present and remains in the waiting room. OR (b) when the intervention is delivered, the pregnant woman and her partner are separated (ie, do not sit together/see/hear one another) and receive different counselling content.
	Shared presence	(3) *Both dyad partners* are invited to the intervention, and *both dyad partners* are present when it is delivered.	A pregnant woman is invited to a maternal counselling appointment. Her partner is invited to accompany her to the counselling appointment and is present in the same room while the pregnant woman receives the intervention (eg, counseling advice).

Scenario 2, the dyadic partial presence, where a dyad partner sits in the waiting room, may seem similar to solo presence (scenario 1). However, we argue that inviting both dyad partners might elicit mutual awareness and shared appraisals that are distinct from solo presence. The dyad partner’s awareness of an intervention being delivered, and the target person’s awareness of their partner’s proximity, might influence post-session interactions and improved communication (eg, debriefing), initiate shared goal discussions, or signal partner availability—processes that differentiate it from a purely individual session. These interactions may in turn potentially trigger dyadic mechanisms like communal coping^15^ or shared reality,^16^ facilitating goal-pursuit and relationship well-being. For example, recent research has shown that more confirming communication (ie, warm and validating messages) was associated with more shared physical activity through enhanced shared physical activity appraisals.^17^ Likewise, work on communal coping in chronic illness demonstrates that when a partner perceives the health concern as “our problem,” the dyad engages in more supportive and collaborative behaviors, which in turn enhance behavior change.^18^

Moreover, shared presence during the session (ie, scenario 3) may activate distinct psychological and physiological processes. As posited by social baseline theory,^19^ mere proximity can reduce cognitive and emotional load by signaling social support. Opportunities for physical touch (eg, holding hands) have been shown to reduce stress.^20^ Taken together, there are several plausible mechanisms indicating that even passive forms of dyad partner presence may influence outcomes through dyadic pathways. While these theoretical implications require empirical validation, the DYADIC meta-framework proposes that dyadic interventions can vary meaningfully based on who is invited and who is present during the intervention delivery, even before considering what is done or how change is achieved.

### Dimension 2: What is done?

The second dimension of the DYADIC meta-framework addresses the intervention content—the BCTs used. BCTs are the “active ingredients” of a behavior change intervention and have been defined as “observable, replicable, and irreducible components of an intervention designed to alter or redirect causal processes that regulate behavior” (p. 82).^21^ In the context of a dyadic intervention, the question, “What is done?,” concerns whether the intervention content includes individual or dyadic BCTs.

To distinguish between individual and dyadic BCTs, consider the following 3 examples: Two best friends are instructed to plan how they will both be more physically active,^22^ a person who smokes is asked to consider the health risks their behavior poses for their roommate, or a buddy is advised to send text messages to another person to remind them of their dietary goals. Would all 3 of these techniques be considered a dyadic BCT and if so, why?

A compendium of *dyadic behavior change techniques (DBCTs)* has characterized the content of dyadic behavior change interventions in couples.^14^^,^^23^ A DBCT is defined as an “observable and replicable intervention technique that explicitly involves any form of interaction with, or clear reference to, a non-professional dyad partner to change behavior” (p. 6)^14^ and specifies “who does what for whom” during an intervention and subsequent implementation. DBCTs are classified into 2 broad types: joint and crossover.

Building on this compendium, the DYADIC meta-framework considers a BCT to be a DBCT if it meets one of the following 2 conditions (Table 2):

**Table 2:** Individual and dyadic operationalizations of dimension 2, “What is done?.”

	Labels	Operational definitions	Example
**Individual**	Individual BCT	(1) The BCT involves only *1 member of the dyad* (target person or dyad partner). AND (2) The BCT does not involve an intended interaction between both dyad partners or a clear reference to the dyad partner.	An individual is instructed to set a goal to improve their physical activity.
**Dyadic**	Dyadic cross-over BCT	(1) The DBCT is targeted at 1 dyad partner and explicitly involves an (a) intended interaction between both members of the dyad at a subsequent timepoint (eg, when at home), OR (b) a clear reference to the other dyad partner.	A buddy is instructed to send text messages to a friend to remind them of their smoking cessation goal. A person is instructed to acknowledge the health risk their smoking poses for their roommate.
	Dyadic joint BCT	(2) The DBCT involves *both dyad partners* interacting. This requires that both dyad partners are not only present at the delivery of the BCT, but also actively perform the task together.	Two adolescent friends are instructed to jointly plan how to both be more physically active.

Abbreviations: BCT, behavior change technique; DBCT, dyadic behavior change technique [14].

First, *joint techniques* require both dyad partners to be present *and* to actively perform the task together during intervention delivery. Examples include jointly planning a physical activity routine for them as a dyad or setting a shared goal for 1 dyad partner, or jointly recognizing risks for 1 dyad partner’s health. Mere shared presence as defined in Dimension 1 is not sufficient, there must be active collaboration.

Second, *crossover techniques* involve 1 dyad partner receiving an instruction that either: (1) prompts an intended interaction with the other dyad partner at a subsequent time point (eg, providing feedback on goal progress, providing support), or (2) includes a clear reference to the other partner (eg, considering health risks of the dyad partner; reflecting on how one’s own behavior affects the other). Only one partner needs to be present for delivery, but the task must explicitly link to the other member of the dyad. Using this logic, all 3 examples mentioned earlier are DBCTs: The first is a joint technique; the second and third are crossover techniques.

By contrast, a BCT is classified as *individual BCT* if it meets *both* of the following conditions (Table 2): The BCT involves only one member of the dyad (target person or dyad partner) and it does *not* involve any instruction to interact with or refer to the other dyad partner. Examples of individual BCTs involve setting a goal for oneself, engaging in self-monitoring, or planning one’s own behavior. Importantly, if both dyad partners receive an individual BCT in parallel (with or without shared presence), this does *not* qualify as a DBCT.

To ensure consistency, we classify BCTs based on the specified intervention protocol, not how the intervention is implemented in practice. If a person is instructed to create their own physical activity plan, but later chooses to do so collaboratively with a dyad partner, this remains an individual BCT because the joint element was not part of the original instruction. It is also important to note that any given intervention may contain a mix of dyadic and individual BCTs, directed at one or both dyad partners. As soon as at least 1 DBCT is included, the intervention content qualifies as dyadic.

### Dimension 3: How does it work?

The third dimension of the DYADIC meta-framework addresses *how* dyadic interventions bring about behavior change. Interventions operate through a mechanistic process involving 2 stages: First, intervention techniques are used to elicit changes in a targeted construct (or set of constructs); second, the elicited change in the construct(s) leads to the desired change in behavior.^24^ These targeted constructs are often referred to as mechanisms of actions (*MoAs*), defined as a psychological, physical or social process or other theoretical construct that is altered or stimulated by BCTs and that influences or catalyzes a change in behavior.^25^^,^^26^

Existing frameworks have made efforts to identify MoAs,^27^ but they typically assume implicitly or explicitly that these mechanisms operate at the individual level, by focusing on, for example, a person’s belief about themselves, the health issue, or the health behavior in question. This approach is consistent with the intrapersonal focus that underlies most if not all health behavior theories.^28^ Yet, with the emergence of dyadic interventions, there is an opportunity to consider the value of distinguishing between MoAs that operate at the individual or the dyadic level. The DYADIC meta-framework can help with broadening the focus from individual to dyadic MoAs.

Consider a program where a person with type-2 diabetes receives reminders from their adult child to adhere to dietary goals. This program could target various mechanisms: increased commitment by the person with diabetes, greater confidence in their success (ie, self-efficacy), or engagement in additional supportive actions by the adult child. The question is: which of these processes are individual and which are dyadic MoAs?

According to the DYADIC meta-framework (Table 3), *individual MoAs* refer to processes occurring within an individual person that influence one’s own behavior. These can be organized into 2 broad classes of constructs: First, *beliefs about the self* encompass thoughts and feelings regarding one’s own attitudes, motivation, or capability (eg, affective attitudes; autonomous motivation; self-efficacy). Second, *self-directed actions* comprise actions people undertake to modify their own behavior (eg, planning, self-monitoring). Although these MoAs operate at the individual level, they may influence not only the person’s own behavior but potentially that of their dyad partner.

**Table 3:** Individual and dyadic operationalizations of dimension 3, “How does it work?.”

	Labels	Operational definitions	Example
**Individual**	Individual belief MoA	(1) Thoughts and feelings people hold regarding their own attitudes, motivations, or capabilities regarding a behavior.	A person’s perception of their own self-efficacy to eat more healthily.
	Individual action MoA	(2) Actions people undertake to modify their own beliefs or behavior.	Self-monitoring of own eating of 5 portions of fruits and vegetables.
**Dyadic**	Dyadic belief MoA	(1) People’s thoughts and feelings about their dyad partner’s attitudes, motivations, or capabilities regarding a behavior.	An adult daughter’s perception of their parent’s self-efficacy to eat more healthily.
	Dyadic action MoA	(2) Actions people undertake to modify a dyad partner’s behavior.	The provision of social support from one family member to another.
	Dyadic shared beliefs MoA	(3) (a) Dyad partners’ beliefs or feelings about the dyad or the relationship OR (b) Any individual beliefs or feelings of both dyad partners that are conceptualized/operationalized at the dyad level (eg, sum, means, differences)	(a) Dyadic efficacy that 2 siblings can work together toward their weight loss. (b) A parent and child’s average perception of their (individual) self-efficacy to eat more healthily on average, or their average closeness.
	Dyadic joint action MoA	(4) (a) The dyad’s joint actions for the dyad OR (b) Any actions of both dyad partners that are conceptualized/operationalized at the dyad level (eg, sum, means, differences)	(a) Cooperative actions in 2 family members. (b) Discrepancy in parent’s and children’s provision of social support to each other.

Abbreviation: MoA, mechanism of action.

*Dyadic MoAs*, by contrast, capture processes between 2 dyad partners that can affect either one dyad partner’s behavior or the dyad’s behavior. We organize these into 4 classes (Table 3). The first class, *beliefs about the other dyad partner* (ie, what I believe about you), refers to thoughts and feelings about the dyad partner’s attitudes, motivations, or capabilities (eg, “I believe my partner is capable of managing their health”). The second class, *actions for the dyad partner or the dyad* (ie, what I do to facilitate your or our behavior change), comprises actions that one dyad partner undertakes to modify the behavior of the other dyad partner or the dyad, such as providing support, exerting social control responding to the partner’s needs (eg, “I support my partner in managing their health”). These actions may be visible or invisible to the dyad partner (eg, invisible support).^29^ The third class, *shared beliefs about the dyad* (ie, what we believe about us), is comprised the dyad’s beliefs about the dyad, such as dyadic efficacy (eg, “We believe we can manage this behavior change together”) or people’s thoughts or feelings about their relationship (eg, closeness; interdependence). This might take the form of seeing one’s dyad partner as responsive or reporting a high degree of commitment to one’s relationship. The fourth class, *joint actions for the dyad* (ie, what we do to facilitate our behavior change), encompasses the dyad’s actions for the dyad and may involve collaborative planning, joint preparation, or cooperative action—processes central to communal coping.^30^

Although dyadic MoAs involve processes between 2 partners, they are often assessed from the perspective of 1 partner. When both dyad partners’ beliefs or behavior are assessed, the MoAs can be operationalized at the dyad level by computing: (1) averages of individual beliefs or actions, to capture shared levels (eg, dyad’s average self-efficacy toward own behavior); or (2) discrepancies to capture degree of agreement or disagreement (eg, differences in dyad partners’ self-efficacy).^31^^,^^32^ This approach can be applied to all classes of individual and dyadic MoAs, rendering them shared beliefs about the dyad (eg, dyad’s average or discrepancies in relationship quality) or joint actions for the dyad (eg, dyad’s average or discrepancies in self-monitoring). This distinction allows researchers to shift from asking *how individuals differ* (eg, “Do supportive partners elicit better outcomes?”) to *how dyads differ* (eg, “Do more responsive dyads show greater behavior change?”). Importantly, using dyadic analysis methods such as the APIM model^31^ or testing interactions between partners does not, by itself, render an individual MoA dyadic unless criteria are otherwise met. These approaches are valuable for examining interdependencies between partners in the MoA-outcome link, for instance, how one person’s individual intention or self-efficacy relates to their partner’s individual behavior.^33^ Thus, while the APIM can reveal associations between individual processes across people and illuminate spillover effects in individual interventions, the underlying constructs remain conceptually individual.

Although MoAs and intervention techniques are often considered at corresponding levels, both individual and dyadic MoAs can be targeted by either BCTs or DBCTs. For instance, an individual MoA like self-efficacy may be targeted by an individual BCT such as behavioral practice^34^ or a DBCT such as reviewing past mastery experiences with the partner.^23^ Likewise, a dyadic MoA such as seeking support can be activated through a DBCT (eg, identifying support needs of one’s partner^23^) or an individual BCT (eg, action planning^34^).

### Dimension 4: What are the outcomes?

Once an MoA has been modified, it is assumed to lead to a desired change in the intervention outcome. All classes of MoAs introduced in the previous dimension can potentially elicit outcomes at either the individual or dyadic level. As a final step in clarifying what makes an intervention dyadic, the DYADIC meta-framework asks: What are the intended outcomes of the intervention?

A common distinction in clinical trials is between primary and secondary outcomes. Primary outcomes are defined as critical for establishing effectiveness of a treatment—for instance, physical activity in an intervention designed to increase physical activity levels. Secondary outcomes are either indicative of additional beneficial effects of the treatment (eg, improved body composition) or help illuminate the intervention’s MoAs (eg, changes in self-efficacy).^35^ We focus on behaviors as primary outcomes, but the DYADIC meta-framework is applicable to outcomes beyond behavior (eg, mental health, relationship well-being).

Outcomes can be conceptualized at the individual or dyadic level. For example, in a rehabilitation program targeting patient A after a myocardial infarction, the primary aim might be to increase patient A’s physical activity. But what if the program instead aimed for patient A and their partner to begin exercising together, or for *both* to reach 150-300 minutes of moderate-to-vigorous physical activity (MVPA) per week? Should these outcomes be classified as individual or dyadic, and what determines that classification?

According to the DYADIC meta-framework, an outcome is classified as *individual* when the focus is solely on the behavior of 1 dyad partner. The guiding research question is: Does the intervention change the [target] person’s behavior? The behavior is enacted independently and does not specifically require joint engagement or coordination (see below).

By contrast, an outcome of an intervention is considered *dyadic* if it meets any one of the following 4 conditions (Table 4):

**Table 4:** Individual and dyadic operationalizations of dimension 4, “What is the outcome?.”

	Labels		Operational definitions	Example
**Individual**	Individual outcome	(1) The behavioral outcome of *1 dyad partner* is the focus of the intervention’s effect. It is enacted independently, not requiring joint engagement or coordination between dyad partners.		A behavior change program aims to improve a cardiac patient’s physical activity and healthy nutrition.
**Dyadic**	Parallel outcome	(1) The behavioral outcome of *each dyad partner* separately is of interest. It does not require joint engagement or coordination, and is not operationalized at the dyad level.		An intervention aims to increase the individual physical activity of a patient, but as a secondary outcome the spouse’s individual physical activity is also assessed and evaluated separately.
	Dyad-level outcome	(2) Any behavioral outcome of both dyad partners that is *operationalized at the dyad level* (eg, shared success, synchrony, similarity, dyad-level sum/mean, or discrepancies).		An intervention aiming to achieve shared success in friend dyads in maintaining smoking abstinence across 6 months, or greater synchrony or similarity in amount of cigarettes smoked.
	Coordinated outcome	(3) The behavioral outcome of both dyad partners that requires coordination between dyad partners and/or a shared agreement of trying to achieve a shared goal.		An intervention with parent dyads aims to achieve that both dyad partners adhere to meeting 150 minutes of moderate-to-vigorous physical activity per week through coordination (eg, 1 parent watches the children while the other exercises and vice versa).
	Joint outcome	(4) The behavioral outcome requires joint engagement in the behavior at the same time and place (ie, with physical or virtual co-presence of both dyad partners).		An intervention aims to increase joint protective sexual behaviors in couples.

First, an outcome is classified as a *parallel outcome* when the focus is on the individual behavior of *each* dyad partner. Thus, the question becomes: Does the intervention change dyad partner A’s behavior, and does it also change dyad partner B’s behavior? For instance, in an intervention designed to increase patient A’s physical activity, both patient A and their spouse may engage in dyadic planning.^36^ While the intervention targets patient A’s behavior as the primary outcome, the extent to which the spouse’s physical activity improves may also be of interest as a parallel (secondary) outcome. Here the focus is on each dyad partner’s individual physical activity behavior as defined as an individual outcome above, but it is essential that both dyad partner’s behaviors are considered.

Second, analogous to MoAs focusing on the level of the dyad (see Dimension 3), *dyad-level outcomes* can be derived by aggregating or comparing both dyad partners’ individual behaviors. The behavior of interest is the individual behavior of both partners, similar to parallel outcomes, but the dyad is treated as the unit of analysis. Examples include:

**Shared success or failure:** This outcome refers to whether both dyad members meet (or fail to meet) a predefined behavioral target together. Success or failure is evaluated at the level of the dyad rather than the individual, meaning that the entire dyad is considered successful only if both members succeed. For example, in an intervention promoting smoking cessation among friend dyads, the outcome may be defined as shared success only if *both* friends remain abstinent from smoking for 6 months. If just 1 partner succeeds, the dyad is classified as not successful.

**Behavioral synchrony:** Behavioral synchrony refers to the temporal alignment or coordination of time-varying behaviors or states between dyad partners.^37^ For example, in an intervention to reduce sedentary behavior, researchers may examine whether the 2 partners increasingly engage in physical activity at the same times during the day.

**Similarity or dissimilarity in behaviors:** This outcome captures the degree of difference or similarity between the behaviors of the 2 dyad partners, regardless of whether they occur at the same time. In a physical activity intervention for romantic partners, researchers may examine whether the difference in daily step counts between partners decreases over time, indicating increased behavioral similarity, whereas an increase over time would indicate behavioral dissimilarity.

**Summative or average outcomes:** These outcomes aggregate both partners’ data into a single value, reflecting the dyad’s overall behavioral performance, status or functioning rather than their alignment. For example, a couple’s quality of life might be assessed as the average of both partners’ well-being scores, their activity level as the combined sum of daily step counts, or their socioeconomic status as total household income. This list of examples is illustrative rather than exhaustive, and additional dyad-level outcomes may emerge depending on context and focus.

Third, *coordinated outcomes* are enacted independently by *each* dyad partner, but require coordination and/or mutual agreement to successfully achieve a shared goal. For example, one partner might agree to watch the children while the other exercises, and then vice versa—allowing both to reach the goal of 150 minutes of MVPA per week. Although performed separately, the behavior change depends on explicit coordination between partners but does not have to be enacted together.

Finally, *joint outcomes* require joint engagement in a behavior by both dyad partners at the same time and place (ie, physical or virtual shared presence). That is, the behavior is enacted *together*. Examples include practicing protective sexual behaviors or exercising together at the same time and (virtual) place.

Although it is often recommended to limit the number of outcomes to maintain statistical clarity,^38^ dyadic intervention studies may benefit from a broader focus, examining both individual and dyadic outcomes. This dual approach can provide a more nuanced picture of how interventions operate within relational contexts and how effects may spread across partners.

## Discussion

The DYADIC meta-framework was developed to provide a structured way to describe key features of dyadic behavior change interventions. It delineates 4 core dimensions—who is present, what is done, how does it work, and what is the outcome—each capturing essential ways dyadic interventions can differ (for a full overview see Table S1). By specifying these features, the meta-framework allows for more precise descriptions and comparisons of interventions involving any dyad partner (eg, romantic partner, friend, family member, etc) in an intervention that would otherwise be grouped under the broad label of “dyadic.”

### Combining intervention components across dimensions

The meta-framework demonstrates that there is no singular form of a dyadic intervention. Instead, a range of possible combinations exist, depending on how individual and dyadic components are integrated across the 4 dimensions. At one end of the spectrum, an intervention may be largely individual with only 1 dyadic element (eg, a college student attending an intervention session receives individual BCTs designed to target individual MoAs to reduce alcohol consumption, but as a dyadic outcome their roommate’s behavior is also of interest). At the other end, all characteristics are dyadic. For example, both dyad partners are present, receive DBCTs designed to elicit dyadic MoAs with the goal of promoting joint engagement of the behavior. Crucially, an intervention is considered “dyadic” if at least 1 dimension involves a dyadic operationalization. This raises the conceptual question of what makes an intervention more or less dyadic—whether it is the number of dyadic elements, their relative importance, or how they are combined. Counting how many dimensions of the framework are operationalized as dyadic may provide a rough indicator of an intervention’s “degree of dyadicness,” but it does not account for the potential differences in relative weight across dimensions. Future research will need to determine the importance of number, proportion and configuration of dyadic dimensions for intervention effectiveness.

Its flexibility allows the framework to go beyond a binary classification of individual vs. dyadic intervention and instead identifies the features that systematically render an intervention dyadic (or not). It also reveals that even individual interventions may include implicit dyadic elements (eg, reliance on partner feedback or social influence)—elements that the meta-framework helps make explicit.

The DYADIC meta-framework also helps to clearly distinguish between different intervention conditions. For instance, in the dyadic condition of the DYACTIC trial,^7^ both partners were present during delivery, both received individual and dyadic BCTs, targeting both individual (eg, self-monitoring) and dyadic (eg, social support) MoAs, with the target person’s behavior as the primary outcome. In contrast, the individual condition in this trial involved shared presence but utilized only individual BCTs to target individual (eg, action control) MoAs to enhance the target person’s behavior as the primary outcome.

Not all combinations of categories across the dimensions are logically possible (eg, joint DBCTs require both partners to be present), and some combinations may be more commonly used than others. To date, some combinations have received more empirical attention than others. For instance, individual MoAs such as autonomous motivation are established predictors of individual behavior change.^39^ While findings show that own beliefs relate to the other or the dyad’s behavior,^32^^,^^33^ evidence regarding the impact of dyadic MoAs remains underexplored. Similarly, links between individual BCTs and MoAs have progressed in recent years,^26^ while links between DBCTs and MoAs are less well established. Recent resources^14^^,^^23^ hypothesize connections between DBCTs and both individual and dyadic MoAs, but more empirical testing is needed.

The DYADIC meta-framework complements existing classification systems such as the Behavior Change Intervention Ontology,^40^ which detail content, delivery, and population characteristics but do not systematically capture the dyadic structure of interventions. These systems remain useful for describing certain features of dyadic interventions, for example, modes of delivery,^41^ the individual BCTs employed^34^ or MoAs.^27^ The meta-framework also extends work in the dyadic field, such as the Compendium of DBCTs.^14^^,^^23^ While existing intervention prototypes^13^^,^^14^ categorize broad intervention types (eg, “mere presence”), the DYADIC framework breaks down the components underlying these prototypes, allowing for more detailed description, coding and theory-building. It offers a common language for describing who is present, what is done (BCTs, DBCTs), how it works (MoAs), and what the intended outcomes are—all at both the individual and dyadic level. The DBCT Compendium v2.0^23^ (www.dbctcompendium.com) remains a key resource for selecting or describing DBCTs; the meta-framework expands this by helping identify other features beyond intervention content.

### Flexibility of the meta-framework

The DYADIC meta-framework is adaptable to diverse intervention contexts, including in-person, remote, asynchronous, or hybrid formats. For example, a partner’s presence during an online session still qualifies as shared presence. Interventions often vary across sessions and dimensions; for example, some sessions may include only the target person, while others involve both dyad partners. The framework encourages detailed reporting of such distinctions.

Although most research has focused on romantic partners, the framework is applicable to any informal, nonprofessional dyad with recurring interactions—be it friends, family members, or roommates. We deliberately varied examples across dyad types to illustrate the framework’s versatility. Dyad type may influence feasibility or impact. For instance, involving the romantic partner or a close family member rather than a work colleague may be more effective due to high goal commitment and goal coordination.^42^ Conversely, having a friend rather than a cohabiting dyad partner wait in the waiting room might mobilize an additional source of support, one that would not naturally emerge without shared daily contact (eg, offering encouragement or help finding relevant information, etc). However, it will be important for future work to discern whether these differences reflect fundamental differences between dyads—if they exist—or are better explained by variation in constructs such as closeness or interdependence. Moreover, depending on the dyad’s goal constellation (eg, parallel or shared goals) as proposed by the Transactive Goal Dynamics Theory,^42^ different dyadic intervention features may be particularly appropriate or effective.

In its current form, the meta-framework focuses on 1 identifiable dyad partner and does not accommodate interventions that involve the social environment which can be comprised multiple, relevant close others. Although it may be extendable to these broader social contexts (eg, rotating caregivers), its current emphasis is on dyads as the smallest social unit. We expect the framework to be applicable and relevant to interventions designed to engage with each of the phases of behavior change, from becoming motivated, to regulating action, and then maintaining a new behavior. However, systematic empirical evidence on phase-specific effects of dyadic interventions remains limited and warrants further investigation. While developed with a focus on behavior change, the framework can be applied to interventions targeting coping, mental health, or relationship functioning.^10^^,^^43^ The framework focuses on behavioral outcomes, but these, in turn, may be targeted because they elicit broader, more distal outcomes such as improved quality of life or sustained health. These interventions and their behavioral outcomes may also affect relationship functioning and satisfaction. Ideally, the health and relational outcomes will both prove beneficial, but there may be times when they are competing.^44^ For example, it has been shown that sharing unhealthy behaviors links to more positive relationship functioning.^45^ Mapping how proximal behavioral outcomes connect to broader health and relational goals is important when developing interventions.

### Limitations

The framework does not prescribe which dyadic elements or combinations are most effective. Instead, it facilitates systematic identification, description, and comparison of interventions to inform future empirical work and allow evidence synthesis. For example, categories such as “partial presence” suggest gradations of dyadic involvement that require empirical scrutiny through the conduct of rigorous experimental studies. Researchers should experimentally vary dyadic elements (eg, presence conditions) while keeping the BCTs constant to assess their effects on MoAs and behavior change.

The categories within each dimension may not be exhaustive. The meta-framework offers a starting point for organizing components of dyadic interventions, but should not restrict how we conceptualize dyadic behavior change interventions. Thus, more diverse or nuanced categories are possible. Also, categorizing intervention components as either individual or dyadic can be challenging and they may fall along a continuum. Boundaries between dimensions can blur. For instance, instructing a dyad partner to provide support in everyday life may be considered a DBCT, but it may be difficult to determine whether—and when—it begins to function as MoA driving behavior change. Nonetheless, rather than prescribing 1 correct categorization, the framework promotes careful reflection and transparent reporting, even if categorization is not always straightforward. Finally, the framework does not address which dyad partner (eg, spouse, parent, friend) to involve. These decisions depend on the health context, population, and intervention goals.

### Implications and future directions

The DYADIC meta-framework offers a structured yet flexible foundation for advancing research and practice in dyadic behavior change. By breaking down interventions into 4 core dimensions, it introduces a shared conceptual language that has been largely absent in this field.

For researchers, the framework promotes transparent reporting and offers a systematic approach to intervention design. This, in turn, enables clearer comparisons across studies and sets the stage for empirical testing of how specific dyadic features influence MoAs and outcomes. Importantly, the framework does not prescribe a particular intervention strategy but prompts researchers to make deliberate and theory-informed choices about when and how to involve dyad partners. It is not a checklist but a tool to foster intentionality in design, encouraging researchers to examine how each dimension should be operationalized—individually or dyadically—based on conceptual rationale and, where possible, empirical evidence.

For example, a physical activity intervention targeting adult best friend dyads might aim to increase shared activity or reduce behavioral discrepancies. This decision will shape other choices about which MoAs to target with which BCTs, and whether one or both friends should attend the intervention. By linking each design choice to the overarching goals of the intervention, the framework ensures that dyadic features are not treated as superficial add-ons but as central, active ingredients.

Although not a step-by-step planning tool, the framework can be used alongside established intervention development frameworks such as Intervention Mapping^46^ which emphasizes understanding the health issue and context (eg, whether a child has a chronic health condition), and identifying long-term and more proximal behavioral goals. Within such a planning process, the DYADIC meta-framework can inform decisions about which dyadic features to include, based on the goals of the intervention and the nature of the dyad.

In applied settings, the framework can support clinicians and interventionists in determining whether, when, and how to involve dyad partners and which psychological processes to target at either the individual or dyadic level.

Looking ahead, the DYADIC framework supports a more cumulative and testable science of dyadic behavior change. It can be used to classify existing interventions, identify gaps, and develop novel approaches that reflect the relational nature of behavior change. Over time, this may help determine which dyadic features uniquely or synergistically promote effective change. The framework also holds promise to being adapted to broader or more fluid social systems and applied in domains beyond health behavior, such as coping with chronic illness, caregiving or relationship functioning.

In sum, the DYADIC meta-framework provides a structured yet adaptable approach to conceptualizing, designing, and evaluating dyadic interventions. By articulating who is present, what is done, how it works, and what the outcome is, the framework promotes conceptual clarity and supports the development of more nuanced and ultimately more effective approaches for behavior change within close relationships.

## Supplementary Material

kaaf102_Supplementary_Data
