# Supplementary material for: What makes an intervention dyadic? Introducing the DYADIC meta-framework to Describe Your focAl Dyadic Intervention Components
Source: Ann Behav Med. 2026 Feb 3;60(1):kaaf102. doi: 10.1093/abm/kaaf102 (PMC12865308; doi:10.1093/abm/kaaf102)
Supplement: kaaf102_Supplementary_Data [file kaaf102_supplementary_data.docx]

Table S1. Summary of Dimensions 1 through 4 of the DYADIC Meta-Framework

|  |  | **Operationalization [Label]** | ***Example*** |  | **Operationalization [Label]** | ***Example*** | |
| --- | --- | --- | --- | --- | --- | --- | --- |
|  |  | **Dimension 1, ‘Who is there?’** | | | | |  |
| **Individual** | 1. One member of the dyad (**Target Person** or **Dyad partner**) is invited to the intervention alone and is the only person present when intervention is delivered.   **[Solo presence]** | | *A pregnant woman is invited to a maternal counselling appointment. She receives counselling regarding maternal health behaviors.* | **Dyadic** | 1. **Both dyad partners** are invited to the intervention, but either **the Target Person or Dyad Partner** is the only person present when intervention is delivered.   **[Dyadic partial-presence]** | *A pregnant woman is invited to a maternal counselling appointment. Her partner is invited to accompany her to the counselling appointment, but…*   1. *when the intervention is delivered (e.g. counselling advice, ultrasound), the partner is not present and remains in the waiting room.*   *OR*   1. *when the intervention is delivered, the pregnant woman and her partner are separated (i.e., do not sit together/see/hear one another) and receive different counselling content.* | |
|  |  |  |  |  | 1. **Both dyad partners** are invited to the intervention, and **both dyad partners** are present when it is delivered.   **[Shared presence]** | *A pregnant woman is invited to a maternal counselling appointment. Her partner is invited to accompany her to the counselling appointment and is present in the same room while the pregnant woman receives the intervention (e.g., counseling advice).* | |
| **Dimension 2, ‘What is done?’** | | | | | | | |
| **Individual** | 1. The BCT involves only ​​**one member of the dyad** (target person or dyad partner).   AND   1. The BCT does not involve an intended interaction between both dyad partners or a clear reference to the dyad partner.   **[Individual BCT]** | | *An individual is instructed to set a goal to improve their physical activity.* | **Dyadic** | 1. The DBCT is targeted at one dyad partner and explicitly involves an 2. intended interaction between both members of the dyad at a subsequent timepoint (e.g. when at home),   OR   1. a clear reference to the other dyad partner.   **[Dyadic cross-over BCT]** | 1. *A buddy is instructed to send text messages to a friend to remind them of their smoking cessation goal.* 2. *A person is instructed to acknowledge the health risk their smoking poses for their roommate.* | |
|  |  |  |  |  | 1. The DBCT involves **both dyad partners** interacting. This requires that both dyad partners are not only present at the delivery of the BCT, but also actively perform the task together.   **[Dyadic Joint BCT]** | *Two adolescent friends are instructed to jointly plan how to both be more physically active.* | |

| **Dimension 3, “How does it work?”** | | | | | |  |
| --- | --- | --- | --- | --- | --- | --- |
| **Individual** | 1. Thoughts and feelings people hold regarding their own attitudes, motivations, or capabilities regarding a behavior.   **[Individual belief MOA]** | *A person’s perception of their own self-efficacy to eat more healthily.* | **Dyadic** | 1. People’s thoughts and feelings about their dyad partner’s attitudes, motivations, or capabilities regarding a behavior;   **[Dyadic belief MOA]** | *An adult daughter’s perception of their parent’s self-efficacy to eat more healthily.* | |
|  | 1. Actions people undertake to modify their own beliefs or behavior.   **[Individual action MOA]** | *Self-monitoring of own eating of five portions of fruits and vegetables.* |  | 1. Actions people undertake to modify a dyad partner’s behavior.   **[Dyadic action MOA]** | *The provision of social support from one family member to another.* | |
|  |  |  |  | 1. a) Dyad partners’ beliefs or feelings about the dyad or the relationship   OR  b) Any individual beliefs or feelings of both dyad partners that are conceptualized/ operationalized at the dyad level (e.g., sum, means, differences)  **[Dyadic shared beliefs MoA]** | *a) Dyadic efficacy that two siblings can work together towards their weight loss.*    *b) A parent and child’s average perception of their (individual) self-efficacy to eat more healthily on average, or their average closeness.* | |
|  |  |  |  | 1. a) The dyad’s joint actions for the dyad   OR  b) Any actions of both dyad partners that are conceptualized/ operationalized at the dyad level (e.g., sum, means, differences)  **[Dyadic joint action MoA]** | *a) Cooperative actions in two family members.*    *b) Discrepancy in parent’s and children’s provision of social support to each other.* | |
| **Dimension 4, “What is the outcome?”** | | | | | |  |
| **Individual** | 1. The behavioral outcome of one dyad partner is the focus of the intervention’s effect. It is enacted independently, not requiring joint engagement or coordination between dyad partners. **[Individual outcome]** | *A behavior change program aims to improve a cardiac patient’s physical activity and healthy nutrition.* | **Dyadic** | 1. The behavioral outcome of **each dyad partner** separately is of interest. It does not require joint engagement or coordination, and is not operationalized at the dyad level).   **[Parallel outcome]** | *An intervention aims to increase the individual physical activity of a patient, but as a secondary outcome the spouse’s individual physical activity is also assessed and evaluated separately.* |  |
|  |  |  |  | 1. Any behavioral outcome of both dyad partners that is operationalized at the dyad level (e.g., shared success, synchrony, similarity, dyad-level sum/mean, or discrepancies.   **[Dyad-level outcome]** | *An intervention aiming to achieve shared success in friend dyads in maintaining smoking abstinence across six months, or greater synchrony or similarity in amount of cigarettes smoked.* |  |
|  |  |  |  | 1. The​​​​ behavioral outcome of both dyad partners that requires coordination between dyad partners and/or a shared agreement of trying to achieve a shared goal.   **[Coordinated outcome]** | *An intervention with parent dyads aims to achieve that both dyad partners adhere to meeting 150 minutes of moderate-to-vigorous physical activity per week through coordination (e.g. one parent watches the children while the other exercises and vice versa).* |  |
|  |  |  |  | 1. The behavioral outcome requires joint engagement in the behavior at the same time and place (i.e. with physical or virtual co-presence of both dyad partners).   **[Joint outcome]** | *An intervention aims to increase joint protective sexual behaviors in couples.* |  |

*Note.* BCT = behavior change technique; DBCT = dyadic behavior change technique [15]; MoA = mechanism of action
